# The "New Movement" in Hospital Appeals

**Published:** 1922-12

**Authors:** J. R. Ainsworth-Davis

**Affiliations:** (Controller of the St. Marylebone Associated Hospitals Local Appeal)


### THE "NEW MOVEMENT" IN HOSPITAL APPEALS.

### WHAT THE MARYLEBONE GROUP HAS DONE.

By MAJOR J. R. AINSWORTH-DAVIS, M.A., M.Sc.
(Controller of the St. Marylebone Associated Hospitals Local Appeal).

HOSPITALS were originally instituted for the medical relief of those absolutely destitute, in times when class distinctions were sharply marked. Their maintenance by voluntary contributions, mostly made by wealthy members of the upper and middle classes, gradually became one of our national customs, and in this respect our hospitals differ from those of other countries. Until the great war this system worked reasonably well, occasional deficits and extraordinary charges being met by various forms of appeal. Some stabilisation of finance was also brought about by the establishment of King Edward's Hospital Fund, the Hospital Sunday and Saturday Funds, and the League of Mercy. The last three of these central agencies, together with similar organised efforts, made it abundantly clear that the hospital-using class was not only willing but anxious to give financial aid.

It is a matter of common knowledge that the shard rise in prices and the greatly increased cost of labour resulting from the great war have had a disastrous influence on our voluntary hospitals, some of which have had to sacrifice their securities and even to close wards. At one time it seemed as if State subsidy and State control would be inevitable. Fortunately, a breathing space has been secured, for the Hospitals of London Combined Appeal for " new " money has been sufficiently successful to justify the belief that existing debts will be wiped out. Now is the time, therefore, to decide on our future policy, and to make up our minds whether the voluntary system can be so adapted and reconstituted as to ensure permanent finnacial stability. If not, it must sooner or later be abandoned. The problem is not too easy, especially as many of those who realised their obligations in the past, and gave large help, are now among the " new poor."

### The Wrong Remedy.

Quite a number of persons, however, appear to approve of handing our hospitals over to the State, asserting, with some show of justice, that the financial problem would thereby be solved, and that the burden would be equitably distributed. But in reality such a system would prove more expensive than the voluntary one, less efficient, and almost entirely devoid of the sympathetic human element so conspicuous in voluntary hospitals. It would be more expensive, because talented honorary medical staffs would no longer be available, subscriptions and donations would vanish, and there would be a cessation of legacies and bequests. More important still, the new branch of the Ministry of Health could not be run as a business concern, and would consequently have no incentive to study economy. Any public criticism there might be would have little if any effect, while voluntary hospitals are bound to regard it and take action if and when necessary.

The State system would also be less efficient, for instead of a constant succession of the best medical talent in existence there would be a large—probably unduly large—permanent staff, doubtless quite efficient at first, but gradually getting less so with the passage of years. Here again public criticism would prove useless, and what we may call the Royal Civilian Medical Service would continue to multiply forms and call for reports of ever-increasing complexity.

That the conduct of hospitals should conform to the highest ideals of altruism is the most important thing of all, and hardly any competent observer could seriously maintain that a State-run department would be likely to excel in this direction. In this ultra-scientific age, life is getting more and more machine-like, and a corrective is badly needed. The Bishop of Birmingham recently said, with much truth, that hospitals afford, if not the last, certainly the highest, form of voluntary effort.

### What the Public Does Not Know.

Before offering some suggestions about possible ways of securing adequate financial support of voluntary kind, it may be well to point out that a great many persons do not realise precisely what is done by our hospitals for their benefit. They know, of course, that large numbers of the sick and injured receive treatment, but they do not understand that most of these are not indigent, as they were in the old days, that they are not now the recipients of "alms" or "charity." Class distinctions are blurred if not obliterated, and there is a better standard of living. While medical treatment, thanks to the existence of honorary medical staffs, remains free, all patients contribute to the cost of maintenance according to their means, and do so with willingness, so that the equivocal words "alms" and "charity" disappear, together with the associations attached to them. All that can be said is that the average hospital patient requires some financial help to secure medical treatment of the highest order. Voluntary subscribers and donors supply that help, and those of them who belong to the hospital-using class are fully aware of this, and therefore feel they are entitled—should they become patients—to be helped in their turn.

Even more important than the treatment of patients is the work of training doctors and nurses which continually goes on in hospitals, and obviously benefits the whole community. An individual who refuses to give any financial help to hospitals, on the ground that—bar accidents—he will never be inside one, entirely forgets this fact.

The third kind of hospital activity—medical research—takes the premier place, although it is the least obvious. By its means one disease after another is being fought successfully, prevention is taking the place of cure, and the study of early symptoms—now coming into prominence—will ultimately render less and less frequent the death sentence "too late" which is daily pronounced by multitudes of specialists. Such research is excessively expensive, and its endowment is recommended to the wealthy, in their own interests no less than in those of others, and the remarks which follow mainly have reference to the maintenance of the other kinds of hospital activity.

### The Local Patriotism of London.

From what has been said it is clear that educational propaganda are essential if we are to secure adequate voluntary support for hospitals, and if the facts are widely understood and appreciated that support will undoubtedly be forthcoming. But man is essentially gregarious, and full success cannot be expected unless large numbers of individuals can be induced to work together, actuated by the same motives, and inspired by enthusiasm for a common cause.

In a provincial city or town the hospital problem is comparatively easy of solution, for there is a traditional spirit of "local patriotism." Fellow citizens realise and are proud of their citizenship, and abundant co-operation can be secured for any good object. The conditions in London are cosmopolitan, and it has often been confidently asserted that provincial ideals and methods are inapplicable. After two years' hospital appeal work in a London borough (St. Marylebone) the writer and his fellow workers have obtained practical results which justify a contrary opinion, especially as the work has been done during a period of greatly depressed trade. Some remarks on methods which are proving successful in one borough may prove of general interest.

### The Lessons of the Marylebone Effort.

London is not a single centre of population, but an aggregate of two cities and a number of towns or boroughs which were once topographically distinct, but are now in contact. They have not, however, lost their individuality, but retain their own history and traditions, and possess their own local government. It is quite true that many of those who work in a borough live elsewhere, but this is no less true of large provincial centres. The new movement aims at fostering local patriotism among those whose interests are mainly centred in a particular borough, in the hope that this may ultimately acquire the characteristics that distinguish provincial cities and towns, thus rendering co-operation easy. A full realisation of this aim would ultimately diminish the overlapping and confusion caused by the innumerable appeals made by individual hospitals (as if no others existed). Institutions in the past have not been accustomed to promote conferences for defining their areas of activity and settling on a common policy, though the pressure of circumstances is beginning to operate in this direction.

St. Marylebone, for instance, has realised the necessity for co-operation, and nine of its voluntary hospitals are now associated together in a local appeal, to those who work in the borough. This appeal does not consist in broadcasting the area with all sorts of propagandist matter. Personal interviews, preceded by letters of introduction, are largely relied upon, for without these the essential human touch is altogether lacking. Such interviews afford the opportunity of describing hospital activities, explaining difficulties, and meeting objections. Employers of labour are usually made to realise that by helping their hospitals they are benefiting themselves as well as their employees, for they are promoting the more important functions of those institutions which have already been mentioned, and which concern everyone.

### The Essential Social Side.

Employees, however, as hospital-users, are even more important than the employers, and if the bulk of them could be induced to subscribe regularly, say, 1d. per week per worker on the average, the financial difficulties of hospitals would be much less. But we cannot expect success with " mass collections " made by workers unless the workers have first-hand knowledge of what the hospitals really mean. Such knowledge is gradually being rendered available to those engaged in the business houses of St. Marylebone by inviting them to social and scientific evenings, so far held at the Middlesex Hospital, but later on to take place as well in some of the other voluntary hospitals with which this is associated.

The results of this propagandist method have been very satisfactory. Guests have shown themselves intensely interested in the scientific side of hospital work, and to many of them it has proved no less than a revelation. A desire to extend practical help has been the invariable sequel, and the desire has realised itself.

A St. Marylebone Hospital Association, controlled by a council made up of representatives of those engaged in the business houses of the borough, has now been formally constituted, and the council is considering how best to promote mass collections. The approved plan will probably take the form of enrolling members willing to pay an annual subscription, fixed at a suitable minimum. This would secure *individual recognition*, for there is a human dignity associated with it which is totally absent from the soulless collecting box practice at present in vogue. Members of an association are far more likely to take an interest and pride in their local hospitals than casters of coin into a box on pay-day. And, after having been entertained as guests at one or more of their hospitals, they will realise what is done with their money and how badly it is needed.

Any virtue the methods of appeal described possess entirely depends on their intimate personal character. Without the human touch failure is assured. Those who give money for furthering a particular object are entitled to full knowledge of the nature of that object, and to knowledge enthusiasm must be added. There can be little if any local patriotism in a London borough unless individuals are brought together as human beings, and imbued with worthy aims and lofty ideals. Those who, like the writer, are closely associated with all sorts and conditions of men cannot fail to realise that the good in human nature far outweighs the bad, and adopt unfailing optimism as the only practical rule of life. If the voluntary system has had a defect, especially in London, hitherto, this has been the absence, on the financial side, of the human element that,in the wards,has made a hospital the happiest place in time of sickness. Till the raising of a hospital's income has been so humanised the problem will remain unsolved. Hospitals are not places of business to their patients. They must free themselves of the commercial taint in dealing with their supporters also.

